# Seeing the self through rose-colored glasses: A cross-cultural study of positive illusions using a behavioral approach

**DOI:** 10.1371/journal.pone.0274535

**Published:** 2022-10-05

**Authors:** Hyunji Kim, Hwaryung Lee, Ronda F. Lo, Eunkook M. Suh, Ulrich Schimmack

**Affiliations:** 1 Department of Psychology, Bielefeld University, Bielefeld, Germany; 2 Department of Psychology, Yonsei University, Seoul, South Korea; 3 Department of Psychology, York University, Toronto, Canada; 4 Department of Psychology, University of Toronto, Toronto, Canada; Polytechnic Institute of Coimbra: Instituto Politecnico de Coimbra, PORTUGAL

## Abstract

Previous studies on self-enhancement bias used self-report measures to investigate individual and cultural differences in well-being. In the current research, we took a behavioral approach to analyze positive and negative perception tendencies between European Canadians, Asian Canadians and Koreans. In Study 1 and 2, participants were asked to bet on their expectation of success on a given task and then perform the task. The betting behaviors and actual performance were used to quantify positive and negative perception tendencies. In Study 1, we did not find cultural differences in positive and negative illusions. Positive self-perceptions were also not associated with higher self-reported well-being. In Study 2, we employed the same research design as Study 1, and we included a measure of perceived desirability to examine whether perceived desirability of the performance tasks are related to the two illusions indices. The results from Study 2 replicated the findings from Study 1, and perceived desirability did not influence the results. Our findings suggest that North Americans do not always exhibit more positive self-perceptions than Asians, suggesting that North Americans do not always view the self through rose-colored lenses.

## Introduction

An influential paper by Taylor and Brown [1] posits that people in general perceive themselves positively. These positive self-views are suggested to be adaptive and beneficial for one’s mental health and well-being. Much research has been conducted since then to understand the conditions and consequences of self-enhancement bias. Relatively little research, however, has been conducted to investigate the universality of positive self-views. That is, most research that explored positive self-perception in desirable traits (e.g., kind, attractive) and emotions was conducted in Western cultural contexts [2–4]. Importantly, cultural research has found members of individualistic cultures to show more positive views of the self compared to those from collectivistic cultures, whereas other studies found no cultural differences in positive self-views [2,5]. While the debate on cultural differences in self-enhancement bias is still ongoing, most prior studies used self-report measures to examine one’s positive perception tendencies. Specifically, studies tended to operationalize self-enhancement as the tendency to make overly positive evaluations of their attributes and traits relative to how they see the average person [3].

To the extent that self-report and informant-report measures of traits reflect subjective judgments of the target, we argue that these reports are vulnerable to various biases and distortions (such as social desirability bias and acquiescence bias). An alternative test of self-enhancement would be to examine whether individuals’ behaviors are influenced by positive self-perception bias by including objective criteria in the measurement of self-enhancement bias [6,7].

In the current study, we used the willingness to bet paradigm as a behavioral measure of self-enhancement. Before performing a broad collection of tasks, participants were asked to bet on succeeding or not succeeding and could win money depending on their betting behaviors and performance. An advantage of this approach is that money is a universally valued resource. Thus, willingness to bet is ideally suited for cross-cultural comparisons of self-enhancement. As well, we looked at a different measure of positive self-perception bias, specifically we focus on self-serving bias in inherently nonsocial domains (e.g., mathematics, geography). The question is whether the findings on positive self-perception bias in general traits would extent to perceived ability.

### Positive self-views

Self-enhancement bias, also called positive illusions, refers to the tendency to perceive oneself as overly positive. Self-enhancement bias is a particularly important psychological process that influences people’s thoughts and behaviors. Indeed, people tend to perceive themselves as better than others on a variety of desirable traits and skills and quickly accept favorable information about themselves but not unfavorable information [3,8]. For example, most respondents rated themselves above average on positive attributes and personality traits (e.g., intelligence, driving ability, charisma) [1,9–11] and performance levels [12]. Self-enhancement has been also associated with betting decisions; that is, people generally believed they are above average, were likely to bet money on themselves and believed their self-enhanced estimates to be accurate [13]. It is important to point out that self-enhancement in Williams and Gilovich’s study was measured with the above-average method and betting was used to measure people’s commitments towards their self-assessments.

Furthermore, Taylor and Brown [1] suggested that most well-adjusted people show unrealistic optimism and self-enhancement as an adaptive strategy to maintain positive self-worth (see also [14]). A more recent paper has conducted a series of laboratory and field studies to examine the (mal)adaptiveness of self-enhancement [15]. Humberg and colleagues [15] concluded that their findings mostly support the notion, “the higher self-perceived intelligence, the better adjusted.” This perspective that self-enhancement is adaptive has been widely accepted in the literature and combined with other theories or fields of study such as romantic relationships [16,17]. However, a major problem in many studies is that self-enhancement and mental health outcomes were based on self-ratings. Specifically, the commonly used self-reported bias indices (e.g., self-other comparison approach, self-insight approach) were found to be confounded with unwanted components in interpersonal perception (the self-other comparison approach is confounded with the target effect) [18] and the reference group effect (the confounding effect in which individuals’ responses to self-report questionnaires are based on respondents’ relative level to a salient comparison group than their actual standing on the variable) [19]. For example, the self-other comparison method (viewing oneself more positively than one views others) is confounded with the target effect (i.e., the extent to which the target is perceived positively or negatively by others). Individuals with a high target effect (i.e., people positively seen by others) tended to also show high self-enhancement bias, thus being erroneously classified as self-enhancers. Some studies avoided this problem by using informant-ratings of mental health and well-being as outcomes. These studies often found no benefits of self-enhancement bias [20,21]. A problem with this approach is that informant-ratings may fail to capture valid benefits of self-enhancement. That is, informants may not have enough relevant information to judge the target’s self-enhancing tendencies or standing on psychological factors (e.g., well-being). As well, informants are also subject to biased judgments (e.g., friendship bias).

To address this concern, we used a behavioral measure of self-enhancement and examined whether this measure of self-enhancement predicts self-ratings of well-being in the current research. Specifically, participants were asked to bet on their performance in a variety of domains (e.g., intellect, physical strength). Participants with a general tendency to exaggerate their abilities should overestimate the probability of winning and bet more often or underestimate the probability of losing. Based on Taylor and Brown’s theory of positive illusions as normative and beneficial, we expected that participants who bet (on succeeding) more frequently than their actual abilities warranted have higher well-being.

### Cultural findings on positive self-views

Among the fundamental questions which remains controversial is the universality of positive self-views [22,23]. Researchers adopting the Universalist perspective propose that these positive perception biases are cultural universals [5,23]. Sedikides and colleagues posit that individuals in all cultures self-enhance, but that culture determines which characteristics are desirable and personally important [23]. Some evidence supports this notion of finding cultural differences in self-enhancement domains between collectivistic cultures and individualistic cultures. People from collectivistic cultures showed positive perception biases on communal attributes, whereas people from individualistic cultures showed biases on agentic attributes [23,24].

In contrast, researchers adopting the Relativist position argue that the self-enhancement bias is not shared in all cultures [22]. The underlying premise of this approach is that a positive image of the self is more important in individualistic cultures as the tendency of people to value the feeling and expression of positive emotions, whereas collectivistic cultures value modesty and self-effacement as desirable attributes that help maintain social harmony. Other studies have found positive perception biases in personality ratings among European North Americans but not Asians or Asian North Americans [20,25]. At present, this controversy has not been resolved. It may be possible that self-ratings of attributes implicitly reflect cultural norms of confidence or excessive optimism, especially in North American cultures. That is, cross-cultural differences in response styles and use of self-report measures of self-enhancement bias may obscure cultural differences in actual self-views [26].

### Present research

In the current study, we use actual behaviors as the objective criteria to examine the perception tendencies in three cultural groups, European Canadians, Asian Canadians and Koreans, and to examine whether positive illusions have any benefit on well-being.

We introduce an approach to measure perception biases based on signal detection theory. Signal detection theory provides a rational framework within which to understand the decision-making processes in the presence of uncertainty [27]. It has been widely used in many applied settings such as diagnostics of cancer, behavioral economics, and psychology [28]. For example, it has been used to study participant’s perception, where participants are presented with a signal for some proportion of the trials and are required to say whether the signal was present or absent. The advantage of utilizing the framework is that it allows us to examine how perceivers make meaningful distinctions (i.e., detect the signal) from background noise. Specifically, the model can separate a perceiver’s behavior into two components, the hit (i.e., correct detection of the stimulus) and correct rejection rates (i.e., correct detection of the incorrect stimulus).

**Fig 1:**
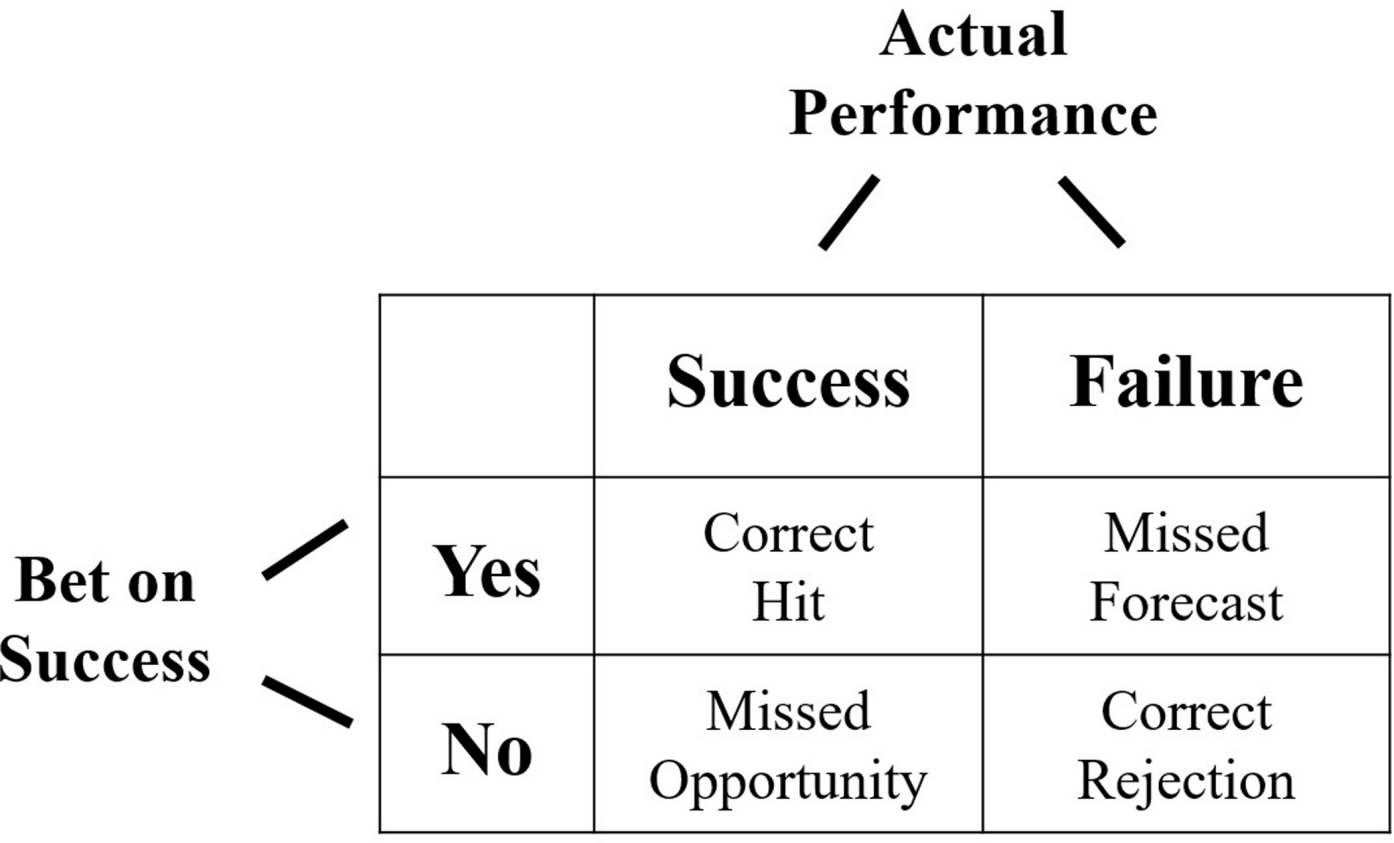
Possible scenarios in the betting task. Positive illusions = Total missed forecasts / (Total missed forecasts + Total correct hits). Negative illusions = Total missed opportunities / (Total missed opportunities + Total correct rejections).

We apply the rational framework to betting situations to determine how an ideal individual (i.e., an individual with realistic perception) would perform given the payoffs and the nature of the task (see Fig 1). The idea is that if an individual has an unrealistic view about their own ability, they should show biases in decision making, such as incorrectly predicting their success or failure in a specific behavioral task. On the other hand, if an individual has a realistic view of their own ability, they are expected to make accurate predictions of their own behavioral performance. Including an objective criterion in the measurement of perception bias can allow for a less biased estimate (e.g., social desirability bias, acquiescence bias) and allows us to make cross-cultural comparisons of positive and negative perception tendencies. Failure to predict success in the tasks will be used as an index of positive illusions, and the likelihood to predict failure will be used as an index of negative illusions. These measures can be calculated for each participant using the formula described below. Positive and negative illusion indices can range from 0 to 1. The closer to 1 the value is, the more likely there is bias. The closer to zero, the less likely that there is bias.

## Study 1

### Methods

#### Ethics statement

The study was approved by the Institutional Ethics Review Board at the University of Toronto. Written informed consents were obtained from all participants prior to the study.

#### Participants

The study consisted of convenience samples of university students from two countries: one hundred seven European Canadian participants (74 women) and 122 Asian Canadians (53 women) were recruited from a university in Canada, and 109 Korean participants (50 women) from a university in South Korea. Canadian participants either received a course credit or monetary compensation (10 CAD) for their participation, and Korean participants received monetary compensation (10,000 Won). The Korean version of the questionnaires had either been validated in previous studies or were translated by two bilingual psychology researchers and one bilingual psychology professor of this paper. Any discrepancies in translation were resolved by consensus between three authors. With no prior data available on the new bias indices, we used a medium effect size (d = .50), a one-tailed alpha of .05 and power of .95 as the criteria to determine the sample size. The estimated sample size was 88 participants per group.

#### Measures

*Betting behavior*. Participants were asked to bet on their expectation of success on ten independent tasks such as solving riddles, spotting differences between two pictures, and answering mathematical questions (see supplementary online material (SOM) for details) and to perform each task regardless of their betting behavior. Expectation of success was measured on a single dichotomous item (1 = yes, 2 = no): Would you bet on winning or not bet on winning?” Actual performance in each task was coded as a dichotomous variable where 1 = success and 0 = failure (see SOM). Before betting on each task, participants were allowed to see a part of the task or try part of the task. The ten tasks were always performed in the same order. The data, and analysis code, and other supplementary materials are available on OSF (https://osf.io/qdxav).

*Life satisfaction*. Life satisfaction judgments were made using the Satisfaction with Life Scale [29] on a scale from 1 (strongly disagree) to 7 (strongly agree). We used the English version for our Canadian participants and the validated Korean version of the Satisfaction with Life Scale for our Korean participants [30]. The first three items (“In most ways my life is close to my ideal”, “The conditions of my life are excellent” and “I am satisfied with my life”) were included as these items have shown better psychometric properties than the last two items [31]. The three items showed good internal consistency in Study 1 (European Canadians, α = .84, Asian Canadians, α = .79, Koreans, α = .86) and Study 2 (European Canadians, α = .87, Asian Canadians, α = .88).

*Liking to bet*. Participants were asked to respond to a single-item statement, “*Do you like to bet*?” with a dichotomous scale (yes or no) in Study 1 and on a 5-point scale from 1 (definitely no) to 5 (definitely yes) in Study 2.

#### Procedure

The study involved three parts: 1) pre-survey, 2) performance task, and 3) post survey. First, participants responded to demographic questions and a set of measures including self-ratings of attributes and life satisfaction. Next, once the participant finished the pre-survey, the experimenter re-entered the room and read the instructions of the performance task to the participant. In the second portion of the study, each participant was accompanied by one or two experimenters, and asked to bet on their expectation of success on ten independent tasks (see SOM for the details of the tasks and evaluation criteria). Participants were told that they could win additional money depending on their betting behavior and actual performance in each task in addition to the compensation for participation. If participants chose to bet on succeeding on the task, whether they won or lost money depended on their actual performance. If they succeeded in the task, they won a dollar. If they did not succeed in the task, they lost a dollar. If participants chose not to bet, the experimenter flipped a coin to let the coin flip decide on whether they won or lost money. If the coin landed heads up, the participant won a dollar, and if the coin landed tails up, the participants lost a dollar. We include the coin-flip to prevent the loss adverse tendencies of individuals since people generally expect losing money to be more painful than expect gaining money to be pleasurable [32]. The purpose was to motivate participants to engage in the task and to prevent them from thinking that it was more rewarding not to bet. In Study 1, participants could win up to 10 dollars in addition to the course credit or monetary compensation. In Study 2, participants were entered into a draw to win a $50 gift card in addition to the course credit. Participants did not receive any feedback on their performance during and after the study, and the procedure made it mostly difficult for participants to guess their performance level on the task. Lastly, participants completed a short questionnaire at the end.

### Results

#### Positive illusions and negative illusions

Data were analyzed using SPSS version 27.0 (SPSS Inc., Chicago, IL) and the R programming language (Version 4.2.0) [33]. We examined cultural group differences using multiple regression analyses with positive and negative illusion indices as the dependent variables.

**Fig 2:**
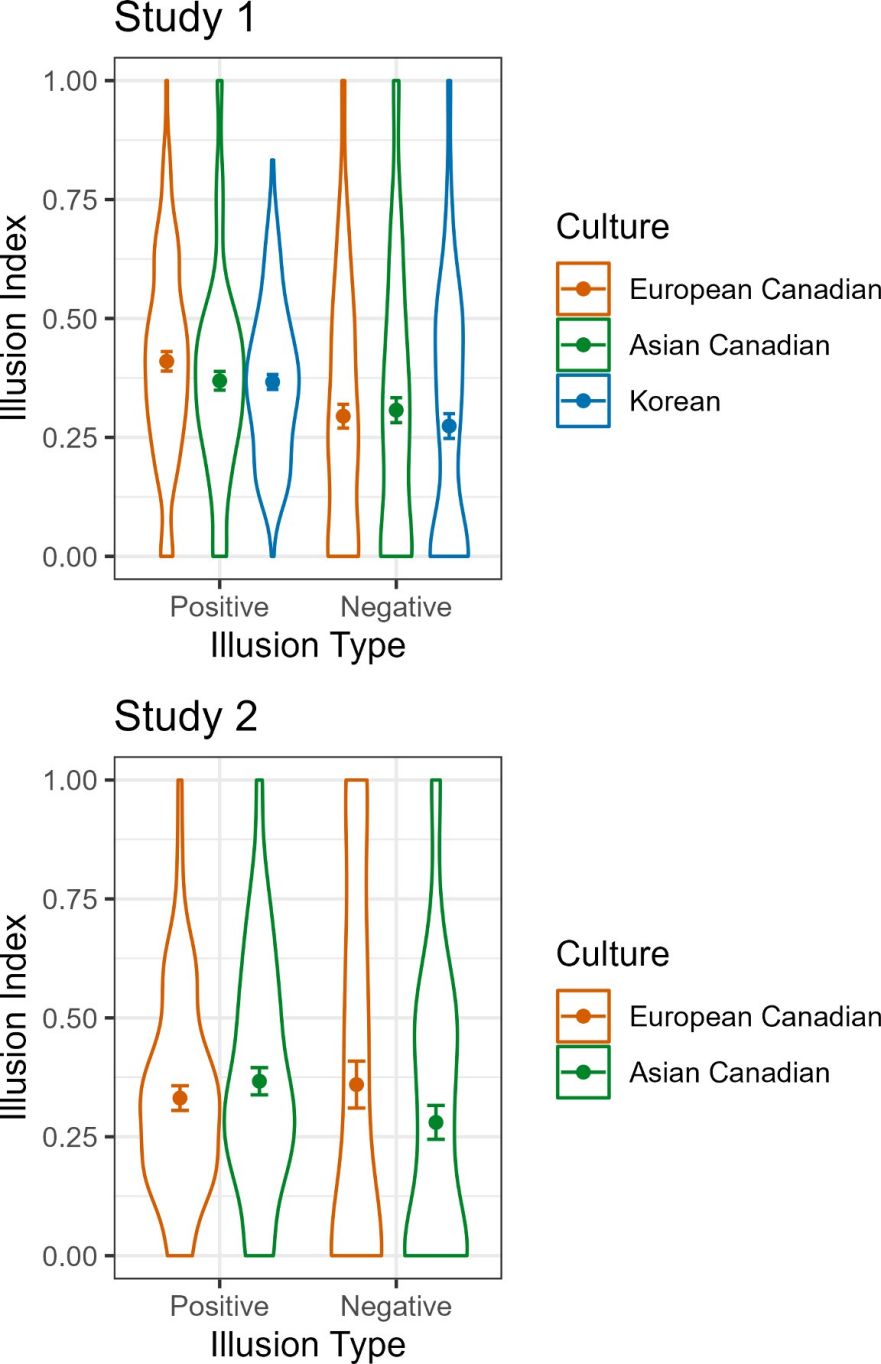
Positive and negative illusion indices. The point estimate and error bars represent the mean and standard error of the mean, respectively. The violin plot represents the distribution of the data.

We first compared the three cultural groups on positive illusions and negative illusions to determine whether European Canadians are more likely to view the self overly positively compared to Asian Canadians and Koreans. Results suggested no cultural differences in positive illusions and negative illusions between the three cultural groups (see Fig 2). European Canadians were not more likely to show positive self-perception or less negative self-perception compared to Asian Canadians and Koreans. Results remained nonsignificant in the post-hoc group comparisons (all *p*s > .09). Next, we entered liking of bets as a covariate. The effect of culture remained non-significant, and no significant effect of covariate was observed.

Additional analyses revealed cultural differences in mean success rates for the tasks across the three cultural groups (see SOM). Thus, we ran additional analyses excluding five tasks (that showed some cultural differences in success rates) and found no cultural differences in positive and negative illusions.

#### Bets, positive illusions and life satisfaction

Next, we examined whether number of bets correlated with life satisfaction. There were no significant relationships between betting times and life satisfaction in the two Canadian cultural groups: European Canadians, *r*(106) = .063, *p* = .53; Asian Canadians, *r*(122) = .06, *p* = .53. Interestingly, Koreans who bet more on succeeding showed higher life satisfaction: *r*(109) = .26, *p* = .006. Lastly, we tested whether having positive illusions was associated with well-being. Positive and negative illusion indices were uncorrelated with life satisfaction respectively across the three groups ranging between -.04 and .12 (all *p*s > .22). Additionally, positive illusion indices were not correlated with negative illusion indices across the three cultural groups: *r*(332) = -.08, *p* = .15.

## Study 2

In Study 2, we extended Study 1 by comparing European Canadians to Asian Canadians and including a measure of perceived desirability to examine whether perceived desirability of the performance tasks is related to the two illusions indices. Some researchers have suggested that people are likely to self-enhance on traits and behaviors that are more desirable or personally important [5,9,34]. Therefore, we tested whether perceived desirability of the performance tasks are related to the illusion indices and whether the relationship between desirability and illusion indices may differ across cultures.

### Methods

#### Ethics statement

The study was approved by the Institutional Ethics Review Board at York University. All participants provided written informed consent before participation.

#### Participants

The final sample consisted of 74 European Canadians and 66 Asian Canadians recruited from a Canadian University. We had more participants who completed the pre-survey and signed up for both the online and in-lab portion of the study. However, we could not complete the in-lab portion of the study (performance task) due to the outbreak of the COVID-19 pandemic.

#### Procedure and measures

The procedure was similar to Study 1, except participants did not get to see part of the task or try out the task before making their bets to reduce the amount of time given to participant to reflect on their abilities. Two tasks (picking up beans with chopsticks and throwing ping pong balls) were excluded from the second study and participants were provided with eight tasks.

*Desirability of performance tasks*. Participants completed the same measures as in Study 1 except we additionally asked participants how desirable it is to do well on each task on a 7-point scale from 1 (very undesirable) to 7 (very desirable). Responses to the eight items per task (“*How desirable would it be for you to do well (succeed) on each task?*”) were averaged to provide a score for overall desirability to perform well (see SOM for the descriptive statistics).

### Results

#### Positive illusions and negative illusions

Consistent with Study 1, European Canadians were not more likely to show more positive illusions or less negative illusions compared to Asian Canadians (see Fig 2). Next, we entered liking of bets as a covariate. The effect of culture remained non-significant, and no significant effect of the covariate was observed. We ran additional analyses excluding four tasks that differed in success rates between the two cultural groups and the results did not change. It is important to mention that the absolute means of the two illusion indices were not close to zero and were similar across the two studies, partly reflecting the difficulty levels of the tasks and suggesting that all cultural groups performed at a similar level.

One limitation from Study 1 is the potential for the desirability of tasks to influence the results. Here we directly tested whether perceived desirability of the performance tasks are related to the two illusion indices and whether the perceived desirability is related with the illusion indices differently across cultures. We performed two multiple regression analyses with culture and perceived desirability as the independent variables, and positive illusions or negative illusions as the dependent variables. Neither main effects nor interaction effects were found for positive illusions and negative illusions (see Table 1).

**Table 1:** Results of the multiple regression analyses in Study 2.

	**Positive Illusions**					
**Model**	**Independent variable**	**B (SE)**	**95% CI**	*t*	*p*	**R²**
1	Culture	0.04 (0.04)	[-0.04, 0.11]	0.92	.36	.01
2	Culture	-0.04 (0.22)	[-0.39, 0.47]	0.17	.86	.05
	Desirability	-0.05 (0.03)	[-0.11, 0.01]	-1.68	.10	
	Culture X Desirability	-0.07 (0.04)	[-0.10, 0.08]	0.15	.88	
	**Negative Illusions**					
	**Independent variable**	**Β (SE)**	**95% CI**	***t***	***p***	**R²**
1	Culture	-0.08 (0.06)	[-0.20, 0.04]	-1.33	.19	.01
2	Culture	-0.36 (0.33)	[-1.02, 0.30]	-1.09	.28	.08
	Desirability	-0.06 (0.05)	[-0.03, 0.15]	1.26	.21	
	Culture X Desirability	0.07 (0.07)	[-0.06, 0.20]	1.02	.31	

Positive illusions (dependent variable): Model 1, *F*(1,136) = 0.84, *p* = .36; Model 2, *F*(3,134) = 2.36, *p* = .07. Negative illusions (dependent variable): Model 1, *F*(1,128) = 1.76, *p* = .19; Model 2, *F*(3,126) = 3.47, *p* = .02.

#### Bets, positive illusions and life satisfaction

Consistent with Study 1, no significant relationship was observed between number of bets and life satisfaction among Asian Canadians, *r*(72) = -.02, p = .89; but the correlation was significant among European Canadians, *r*(66) = .33, p = .008. Lastly, we tested whether having positive illusions is good for one’s well-being. Positive and negative illusion indices were uncorrelated with life satisfaction across the two cultural groups, ranging between -.19 and .05 (all *p*s > .12). Additionally, positive illusion was uncorrelated with negative illusion among Asian Canadians, *r*(72) = -.06, p = .66; and, positive illusion was slightly correlated with negative illusion among European Canadians, *r*(62) = -.25, p = .05. Combined with the results from Study 1, these results suggest that having an illusion of oneself is neither psychologically beneficial nor damaging, and that the two perception biases may be independent of each other, each contributing additively to cognitive outcomes.

## Discussion

Positive illusions are consistent patterns of favorable self-evaluations observed in domains of self-relevant traits and behaviors, including personality traits and abilities [1]. Recent studies have found positive perception biases in emotion judgments [4]. It has been widely observed in many areas of life that people view themselves in overly positive ways to regulate their self-esteem or respond to situational cues. Many studies have focused on the measurements of positive illusions in inherently social domains asking participants to evaluate their personality, and have examined positive illusions in different cultural contexts using self-reports. The question is whether the findings on self-enhancement bias in traits would extend to behaviors, specifically when self-ratings are compared to actual performance. Here, we moved away from assessing self-enhancement using self-reports, focusing instead on behavioral predictions and actual behaviors.

In the current research, we used a behavioral approach to examine cultural differences in positive and negative self-perception tendencies between European Canadians, Asian Canadians, and Koreans, and examined whether having positive illusions are good for well-being. The two illusion indices were defined as the match between behavioral predictions and performance on the tasks. With the inclusion of objective criteria, the positive and negative illusion indices are less likely to be confounded with biases in self-reports (e.g., shared method bias, bias in target effects). Interestingly, we did not find cultural differences in positive and negative illusions between the three cultural groups and did not find associations between the two illusions and well-being in the current research. These patterns were robust across the two cultural samples. Importantly, the absolute values of averaged illusion indices were similar across the cultural groups and two studies, suggesting all individuals may not have shown neither positive nor negative self-perceptions but realistic self-perceptions.

Prior work on self-enhancement bias provided mixed cultural findings; some research found no cultural differences in self-enhancement bias, whereas other studies have observed self-enhancement bias more in the North American cultures than in Asian cultures or only in North American cultures [12,22,23,35,36]. Many of the major findings from this literature measured self-enhancement bias using the above-average method or discrepancy method between self-reported characteristic score and other-reported (or informant-reported) characteristic score.

Although these methods have been suggested as acceptable indices for measuring positive self-perceptions, previous work has shown that these bias indices to be confounded with unwanted components in interpersonal perception [18]. Integrating previous work and the current results, European Canadians compared to Asians may not be more likely to show overly positive self-perceptions in evaluations of one’s own performance, and self-enhancement bias may be more likely to emerge in self-reports of questionnaires. Thus, our research suggests that future cultural work on self-enhancement bias should examine biases using different methods and biases in both social and non-social domains to establish a comprehensive picture of the cultural difference in bias.

The current work also questioned the beneficial effects of positive illusions on well-being [1] and concludes that positive illusions on specific behavioral tasks are not likely to be associated with higher self-reported well-being. Two explanations may account for the null findings. First, the positive association between self-enhancement bias and well-being may only be observed in questionnaires, reflecting shared method bias in self-report questionnaires [20]. Indeed, this positive association was mostly observed in studies that indexed self-enhancement bias via self-ratings of traits and abilities (e.g., social comparison method, above average method) [1,2,36]. As well, the positive association was also mostly observed between the bias measure and self-reported well-being but not informant-reported well-being [20,37–39]. For example, Kim and colleagues [20] included self- and informant-reported well-being and found positive associations between positive illusions and self-reported well-being but not informant-reported well-being. As well, self-enhancers provided positive evaluations of their own mental health but were not viewed by observers, professional clinicians, as having more positive qualities [39]. Some studies have even found positive illusions to be maladaptive [37,38].

Second, given the research design and practice trials, people perhaps were more likely to show realistic perception, that is, realistically evaluate their own ability and make predictions accordingly in the current research. According to the Cognitive-Experiential Self Theory [40], people’s perceptions are guided by two information processing systems: (1) the cognitive system is analytical, slow and deliberate, and more likely to operate through reason for delayed behavior; (2) the experimental system is automatic, emotional and are more likely to operate for immediate behavior. Although both systems can operate independently but also in parallel and influence self-perceptions, it may be possible that in the current research people’s perceptions were likely guided by the cognitive system. If participants had enough time to think about their abilities, they could adjust their predictions based on the practice trials (in Study 1) and detailed instructions to think about their ability before making their predictions. We also note that the absolute values of the two mean illusion indices were similar across different cultural groups and two studies, providing additional support that people in the current research showed realistic perceptions. The numbers may partially reflect the difficulty levels of the tasks and error in judgments. As well, we think that the incentives for accurate perceptions may have influenced everyone, including people who generally view themselves overly positive to show realistic perceptions. Indeed, prior research has found monetary reward to influence people’s culturally valued (or “default”) self-evaluation judgment [12]. Yamagashi and colleagues used the above-average method to evaluate self-enhancement or self-effacement tendencies. In their study, participants were asked to perform a test and to evaluate whether they think their performance was above average or below average after the task. They found that Japanese people were likely to exhibit modest responses or self-effacing tendency and American people to exhibit self-enhancing tendency in the natural environment (with no situational constraints). However, interestingly, the cultural difference disappeared when participants were provided with a monetary reward to make accurate predictions about their performance level. Further investigations are warranted to clarify whether the study design, incentives or operationalization of illusion indices may have resulted in null effects. We suggest that it is important to examine the question using not only self-reports (e.g., above-average method) but using behavioral predictions and objective criteria for the measurement of self-enhancement bias.

We note two limitations of our studies that need to be addressed in future research. First, the difficulty of the behavioral tasks was set from easy to moderate across all cultural groups. However, individuals from the three cultural groups showed discrepancies in success rates suggesting that certain tasks were easier for certain cultural groups. Furthermore, the overall success rate was higher for Koreans suggesting that the task levels were still easy for Korean undergraduate students. Thus, Koreans may have been less likely to be self-critical and more likely to bet on succeeding out of self-confidence (rather out of self-reflection). Thus, future research needs to replicate these findings with different samples and task difficulty levels, preferably using behavioral tasks that show similar levels of success rate across cultural groups comparing different individualistic and collectivistic cultures. However, it is important to note the current results did not change when the analysis was restricted to tasks which showed no or small cultural differences in success rates suggesting that people in general showed similar perceptions of their ability on the various tasks. We also note that it is important to move beyond the prevailing East versus West comparison, and include various cultural groups such as the Arab culture which shows a different profile of interdependence. The Arab culture is characterized by a self-assertive form of interdependence which is distinct from that of either East Asians or Westerners [41]. Thus, it would be important to examine whether Arabs would show patterns of bias analogous to that of East Asians and Westerners to further understand the mechanisms, degree and conditions of self-enhancement bias. For instance, this investigation will provide a clearer picture if the known cultural difference in self-enhancement bias between East Asians and North Americans occurs as a result of the promotion of positive thinking in North American culture, East Asians’ modesty concerns (or even self-effacement tendencies), or interdependence more broadly (i.e., applicable to both Asians and Arabs).

Second, another limitation was the use of dichotomous variables for measurement of illusions and the lack of standard definition for the suggested positive and negative illusion indices. The indices were measured with the frequency of successes or failures and number of bets or no bets. The response in each trial was dichotomous rather than continuous. Theoretically, this is addressed by construing the data as having equal number of observations in each condition [42]. However, in research, it is common to have unequal number of observations across conditions. The distribution of the estimates may also not approximate a continuous distribution. This may result in ceiling or floor effects in situations involving either overly positive or overly negative perceivers. The results did not change when the analyses were repeated including tasks with similar success rates across groups, thus, it is unlikely that the results are due to ceiling effects. In future work, it would be of interest to obtain actual behaviors using other response scales. For example, instead of rating participants’ behaviors dichotomously, behaviors could be viewed as a continuous action. Furthermore, there is no standardized way of operationalizing the numbers for the constructed bias indices. The two illusions indices can range from 0 to 1, but there is no standard approach to interpreting the numbers between 0 and 1. Although this is a limitation, it is not a unique problem of the current research, rather it is a general limitation of all studies using nonparametric measures.

Despite the limitation of the present research, our work has major implications. First, our results suggest that European Canadians do not always view the self overly positive compared to Asian Canadians and Asians. That is, those from individualistic cultures are not merely blinded by rose-colored dreams. It may be the case that situational factors (e.g., incentives, information on the task, amount of time) influence the degree to which an individual views their own characteristics as overly positive. Our results may also suggest that the stark cultural differences in self-enhancement bias are observed in self-reports of self-enhancement bias. That is, North Americans are more likely to show favorable self-ratings of own traits and if there are no situational constraints on the individual (e.g., time influence, incentives). Second, many psychological studies build on Taylor and Brown’s theory of positive illusions, which posits that positive illusions are pervasive in normal human thoughts and are beneficial for mental health [1]. Our results did not provide evidence for an association between positive illusions and well-being. According to our results, positive self-perceptions may reflect shared method bias in studies that use self-reports, and positive self-perceptions are not observed when using objective behavioral criteria. Lastly, most prior research has focused on positive perception biases and relative little attention has been placed on negative perception biases. To the best of our knowledge, the current research is the first to compare both positive and negative illusions in different cultural groups using behavioral predictions and objective criteria for the measurement of self-enhancement bias. The present research adds value to the literature on self-perception and cultural research by conceptualizing illusions in a different way demonstrating that European Canadians compared to Asians are not always more likely to view the self positively, Asians compared to European Canadians are not always likely to view the self negatively, and overly positive self-perception may not be linked to well-being.

## Supporting information

S1 File (DOCX)
