# Supplementary material for: Seeing the self through rose-colored glasses: A cross-cultural study of positive illusions using a behavioral approach
Source: PLoS One. 2022 Oct 5;17(10):e0274535. doi: 10.1371/journal.pone.0274535 (PMC9534404; doi:10.1371/journal.pone.0274535)
Supplement: S1 File — (DOCX) [file pone.0274535.s001.docx]

**Online Supplemental Material**

**Table S1. Task Description.**

| Task | Description | Criteria for Success | Example |
| --- | --- | --- | --- |
| Memory card game | Memorize the placement of five pairs of matching symbol cards for 30 seconds. The cards were then flipped over, and participants were asked to correctly locate two matching cards at a time. | Remembering the locations of all 5 pairs. | *Memory Game: Sesame Street Edition |
| Word | Remember the order of all 8 words correctly. | Remembering the order of all 8 words correctly. | Marvellous, Mystique, Mystery, Morning, Mall, Maroon, Majority, Moon |
| Paragraph | Read a paragraph out loud, correctly, within 30 seconds. | Reading the paragraph out loud within the time limit. | “The furry, brown kiwi does not look much like a bird. It has whiskers like a cat, and its feathers looks like a mouse's fur coat. Rabbits and other animals also have whiskers. The kiwi cannot fly. It can walk almost silently on its soft feet. It lives in the mountain forests of New Zealand.” |
| Riddles | Answer 2 out of 3 riddles correctly. | Solving 2 out of 3 riddles. | Take off my skin and I won't cry, but you will, what am I? Answer: Onion |
| Spotting the difference picture task | Identify 7 differences between two similar pictures within a minute | Identifying at least 7 differences. | *Pictures were given to participants |
| Chopsticks | Move 8 beans from one container to another with chopsticks within 30 seconds. | Moving at least 8 beans from one container to the other. |  |
| Math problems | Solve 3 out of 4 math problems. | Solving 3 out of 4 math problems correctly . | Question. Convert: 6 km 0m 4 cm = ( ) cm  A. 600004 (Answer)  B. 6004  C. 60004  D. 6000004 |
| Throwing ping pong balls | Throw 2 out of 3 ping pong balls into a container. | Successfully throwing 2 out of 3 ping pong balls into the container. |  |
| Countries | Identify 5 countries on a world map. | Identify 4/5 countries. | 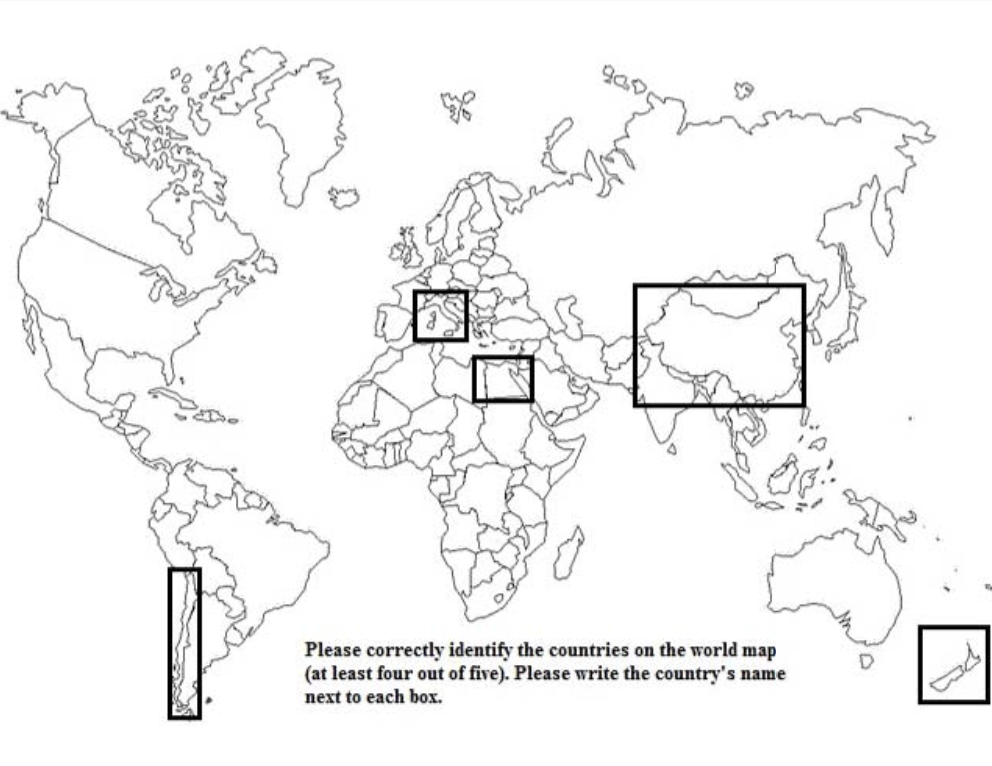 |
| Handgrip | Squeeze a handgrip with the dominant hand for 50 seconds for men and 30 seconds for women. | Successfully squeezing the handgrip for the entire period. |  |

**Text S2. Experimenter Script and Coding.**

In the next part of the study, you will be asked to bet on your expectation of success on each given task, and then asked to perform the task. There are ten different tasks in total, and you have a chance to win 10 dollars (or 10000 Won for Koreans) today. Before performing each task, for example, answering riddles, you will be asked to bet on your expectation of success on the task. Afterwards, you will be asked to perform the task. If you decide to bet on winning that is succeeding the task, and then successfully perform the task, you will win ONE dollar (or 1000 Won). If you decide to bet and fail the task, you will lose one dollar. If you decide not to bet on winning, the experimenter will flip a coin and let the coin decide on winning or losing a dollar. If the coin lands heads up, you will win one dollar, and if the coin lands tails up, you will lose a dollar.

Here is the opportunity to win more money in addition to the credit (or monetary compensation). The difficulty of each task is set to easy to moderate. If you know yourself well, and think you have a higher chance than 50% to succeed on the task, it is better to bet on winning to maximize the money you can make. You will not be losing your own money nor will be giving us your own money in any case. However, please think carefully and try your best to complete each task.

---

Ok let’s start with the first task, which is the memory card game.

1. You will be provided with 5 pairs of picture cards, so 10 cards in total, and asked to REMEMBER the locations of 5 pairs for 30 seconds. First, I will place the cards face up on the table. You will have 30 seconds to remember the locations of the 5 pairs of picture cards. After 30 seconds, I will ask you to turn around and face the wall while I place the cards faced down. Afterwards, you would be asked to turn over EACH PAIR at a time. The goal is to correctly match all 5 pairs of the picture cards. Would you bet on winning or not bet on winning?

□ Bet on winning □ No bet

Coin: □ Heads (+1dollar) □ Tails (-1 dollar)

Performance: □ Succeeded □ Failed #/Time:

1. You will be given 30 seconds to remember the order of 8 words. Afterwards you will be asked to put them in the ascending numerical order using numbers from 1 to 8 (1 = first, 2= second, 8 = last). Would you bet on winning or not bet on winning?

□ Bet on winning □ No bet

Coin: □ Heads (+1dollar) □ Tails (-1 dollar)

Performance: □ Succeeded □ Failed #/Time:

1. You will be asked to use chopsticks to move 8 beans from one container to the other container within 30 seconds. Would you bet on winning or not bet on winning?

□ Bet on winning □ No bet

Coin: □ Heads (+1dollar) □ Tails (-1 dollar)

Performance: □ Succeeded □ Failed #/Time:

1. You will be given three riddles and asked to answer at least 2 correctly. (participants get max of 3-5 minutes) Would you bet on winning or not bet on winning?

□ Bet on winning □ No bet

Coin: □ Heads (+1dollar) □ Tails (-1 dollar)

Performance: □ Succeeded □ Failed #/Time:

1. You will be given two pictures of the same thing and asked to spot at least SEVEN out of 10 differences between the two pictures within 1 minute. Would you bet on winning or not bet on winning?

□ Bet on winning □ No bet

Coin: □ Heads (+1dollar) □ Tails (-1 dollar)

Performance: □ Succeeded □ Failed #/Time:

1. You will be given five mathematical problems and asked to answer at least 3 (including 3) out of five mathematical problems correctly. (max of 5 minutes) Would you bet on winning or not bet on winning?

□ Bet on winning □ No bet

Coin: □ Heads (+1dollar) □ Tails (-1 dollar)

Performance: □ Succeeded □ Failed #/Time:

1. You will be given three ping pong balls and asked to throw at least two balls into the cup (that is 50cm in front of you). Would you bet on winning or not bet on winning?

□ Bet on winning □ No bet

Coin: □ Heads (+1dollar) □ Tails (-1 dollar)

Performance: □ Succeeded □ Failed #/Time:

1. You will be given a paragraph and asked to read it within 15 seconds. Would you bet on winning or not bet on winning?

□ Bet on winning □ No bet

Coin: □ Heads (+1dollar) □ Tails (-1 dollar)

Performance: □ Succeeded □ Failed #/Time:

1. You will be given a picture of the world map and asked to identify the names of at least four out of five countries correctly on the map. (max of 5 minutes) Would you bet on winning or not bet on winning?

□ Bet on winning □ No bet

Coin: □ Heads (+1dollar) □ Tails (-1 dollar)

Performance: □ Succeeded □ Failed #/Time:

1. You will be given a handgrip and asked to squeeze the handgrip with your dominant hand for more than 30 seconds (female) [or 50 seconds (male)]. Would you bet on winning or not bet on winning?

□ Bet on winning □ No bet

Coin: □ Heads (+1dollar) □ Tails (-1 dollar)

Performance: □ Succeeded □ Failed #/Time:

*Note*. Two tasks were not included in Study 2 (moving beans with chopsticks, throwing ping pong balls into a container).

**Table S3. The Success and Betting rates across Three Cultural Groups (Study 1).**

|  | Card | Word | Bean | Riddle | Spot | Math | Throwing | Paragraph | Map | Handgrip |
| --- | --- | --- | --- | --- | --- | --- | --- | --- | --- | --- |
| *Success Rate* |  |  |  |  |  |  |  |  |  |  |
| European Canadians | 64.5 | 47.7 | 26.7 | 52.8 | 42.1 | 44.9 | 24.4 | 70.8 | 42.5 | 50.9 |
| Asian Canadians | 60.7 | 33.1 | 58.2 | 52.5 | 54.1 | 70.5 | 36.4 | 46.7 | 38.0 | 57.9 |
| Korean | 65.7 | 73.4 | 20.2 | 62.4 | 72.5 | 89.0 | 20.2 | 21.1 | 54.1 | 59.6 |
| *Betting Rate* |  |  |  |  |  |  |  |  |  |  |
| European Canadians | 72.0 | 52.3 | 47.2 | 60.7 | 90.7 | 37.4 | 41.1 | 77.4 | 42.1 | 50.0 |
| Asian Canadians | 68.0 | 61.5 | 73.8 | 32.8 | 89.3 | 77.9 | 48.4 | 72.1 | 47.5 | 48.4 |
| Korean | 80.7 | 89.0 | 67.0 | 64.2 | 91.7 | 91.7 | 46.8 | 81.7 | 56.9 | 52.3 |

*Note*. Success Rate (%) is calculated by dividing number of individuals who succeeded in the task by number of individuals who participated in the task. Betting Rate (%) is calculated by dividing number of individuals who bet on succeeding in the task by number of individuals who responded to the betting question. The difficulty was set from easy to moderate based on the subsample of Canadians. This step was necessary to ensure that the decisions are made based on information rather than “gut” feelings. We ran additional analyses with five tasks with smaller differences in success rates (Study 1 - card, riddle, throwing, map, and handgrip). The pattern of the results remained essentially the same and the cultural difference was smaller in magnitude.

**Table S4. Descriptive Statistics of Variables in Study 1.**

|  | Positive Illusions | Negative Illusions | Number of Bets | Life Satisfaction |
| --- | --- | --- | --- | --- |
| European Canadians | .41 (.21) | .30 (.26) | 5.73 (1.56) | 5.05 (1.12) |
| Asian Canadians | .37 (.22) | .31 (.29) | 6.20 (1.73) | 4.74 (1.09) |
| Korean | .37 (.16) | .27 .(27) | 7.21 (1.35) | 4.57 (1.33) |

*Note*. Mean (Standard Deviation).

**Table S5. The Success and Betting Rates across Two Cultural Groups (Study 2).**

|  | Card | Word | Riddle | Spot | Math | Paragraph | Map | Handgrip |
| --- | --- | --- | --- | --- | --- | --- | --- | --- |
| *Success Rate* |  |  |  |  |  |  |  |  |
| European Canadians | 80.3 | 40.9 | 42.4 | 81.8 | 36.4 | 80.3 | 36.9 | 69.7 |
| Asian Canadians | 73.6 | 43.1 | 40.3 | 75.0 | 69.4 | 33.3 | 12.5 | 63.9 |
| *Betting Rate* |  |  |  |  |  |  |  |  |
| European Canadians | 89.4 | 72.7 | 56.1 | 92.4 | 62.1 | 83.3 | 34.8 | 89.2 |
| Asian Canadians | 86.1 | 83.1 | 33.3 | 86.1 | 69.4 | 66.7 | 32.4 | 63.9 |

*Note*. We ran additional analyses with five tasks with smaller differences in success rates (Study 2 - card, word, riddle, spot, handgrip). The pattern of the results remained essentially the same with five tasks.

**Table S6**. **Descriptive Statistics of Variables in Study 2.**

|  | Positive Illusions | Negative Illusions | Number of Bets | Life Satisfaction | Liking to bet | Desirability |
| --- | --- | --- | --- | --- | --- | --- |
| European Canadians | .33 (.21) | .36 (.39) | 5.83 (1.27) | 4.72 (1.34) | 2.45 (1.24) | 5.13 (.91) |
| Asian Canadians | .37 (.24) | .28 (.29) | 5.22 (1.45) | 4.42 (1.18) | 2.67 (1.07) | 4.58 (.85) |

*Note*. Mean (Standard Deviation). Liking to bet: Participants responded to the single item “Do you like to bet” on a 5-point scale from 1 (definitely no) to 5 (definitely yes). Desirability: Participants evaluated how desirable it is to do well on each task on a 7-point scale from 1 (very undesirable) to 7 (very desirable). Responses to eight tasks were averaged to represent the overall desirability score.
